# Oncological safety and short-term aesthetic outcome of extreme oncoplasty for breast cancer

**DOI:** 10.1186/s43046-026-00348-9

**Published:** 2026-03-23

**Authors:** Mohamed Ibrahim Abdel Aziz, Abdullah Ali Ahmed Mohammed, Sameh Mohamed Samir, Mohamed Moustafa Farag

**Affiliations:** Department of GeneralSurgery, Faculty of Medicine, Fayoum University, Fayoum, Egypt

**Keywords:** Extreme oncoplasty, Breast cancer, Aesthetic outcome, Oncological safety

## Abstract

**Background:**

For individuals with breast cancer, oncoplastic breast surgery (OBS), is considered an innovative technique of breast conservative surgery (BCS), has emerged as the recommended substitute for mastectomy. BCS and mastectomy have been shown to have comparable locoregional control and overall survival in randomized trials. In order to ensure oncological safety and improved aesthetic results, Extreme Oncoplasty, a development in the field of oncoplasty, has given hope for patient to achieve ultimate conservation before mastectomy.

**Patients and methods:**

From October 2021 to May 2023, fifty patients fulfilling our eligibility criteria underwent extreme oncoplasty for breast cancer have been included in the current study. Reduction mammoplasty (superomedial, superiolateral and inferior pedicle), upper matrix rotational flap, and Passot’s technique were our adopted surgical techniques. One year following surgery, short-term aesthetic results were objectively assessed using Breast Cancer Conservative Treatment (BCCT) core program.

**Results:**

Our study has included 50 cases with age range from 35 to 61 years (mean 52.28 ± 6.3). 38 patients presented with tumor size more than 5 cm not responding to the neoadjuvant therapy for downsizing and 12 patients presented with multifocal lesions. The total aesthetic results according to the BCCT core software were good in 52% (26 cases), fair in 34% (17 cases) and poor in 14% (7 cases). Our study has reported two cases of recurrence (4%) one year after surgery which were managed via mastectomy.

**Conclusion:**

According to short-term follow-up, extreme oncoplasty is oncologically safe. To provide patients with advanced or multifocal breast cancer with extreme oncoplasty as a viable alternative, large-scale research are needed to validate these first findings.

## Introduction

When compared to mastectomy, breast conservative therapy (BCT) has been shown in many prospective randomized studies to have similar overall and disease-free survival rates. This fact has installed the BCT as the routine management option for early stage breast cancer [1–4]. 

Various oncoplastic techniques for excision and reconstruction have been used in subsequent oncoplastic breast surgery developments, which have further improved the oncologic and aesthetic results [5–7]. Many volume displacement and replacement techniques have been added to the oncoplasty spectrum to enable greater resections with a lower incidence of inadequate margins and revisional procedures [5, 8]. Extreme oncoplasty is one of those innovative oncoplastic concepts which have been introduced in the last years to guarantee breast conservation for patients who, in most physicians’ opinions, warrant a mastectomy [9]. Silverstein advocated this new concept of extreme oncoplasty for his patients presented with multifocal, multicentric tumors, and those presented with a tumor greater than 5 cm. He has reported great results on 66 patients, with 6% undergoing mastectomy and 10% needing re-excision due to insufficient margins [10]. He came to the conclusion that people who originally need mastectomy may benefit from extreme oncoplasty, which has promising oncological and aesthetic outcomes. In the current study, we have chosen to investigate the short-term oncologic and aesthetic results for our patients who fulfill conventional mastectomy recommendations, and were managed with oncoplastic surgical techniques.

## Patients and methods

From October 2021 to May 2023; 50 breast cancer patients fulfilling our eligibility criteria were enrolled in this prospective study. Our multidisciplinary team ( oncoplastic breast surgeons, oncologists, radiologists, and pathologists) discussed the inclusion criteria for each case. They were surgically managed in Fayoum university hospitals with three different volume displacement oncoplastic surgical techniques to enable us to deal with each tumour location achieving good oncological and aesthetic otucomes. Reduction mammoplasty with superiomedial pedicle for tumours located in the lower outer quadrant of the breast, superiolateral pedicle for tumours located in the lower inner quadrant of the breast and inferior pedicle for tumours located in the upper pole of the breast. Upper matrix rotational flap for tumour located in the upper inner breast quadrant and Passot’s technique for tumours located in the lower pole of the breast. Every tumor was removed with measurable safety margins of one centimeter. To overcome the postoperative challenge of radiation therapy over the tumour bed after displacement of the parenchymal flaps, we marked the four sides of the excision cavity and the bed with titanium clips (6 clips) as a landmark for guidance of the adjuvant radiotherapy. This vital operative step, we considered, has facilitated to a far extent the mission of effective delivery of adjuvant radiotherapy.

Mammography and complementary ultrasound were done on biannual basis for follow up of the oncological outcome.

A year following surgery, an objective assessment of aesthetics was conducted using the semi-automated BCCT core software utilizing the same principles of Cordoso et al. [11].

**Fig. 1:**
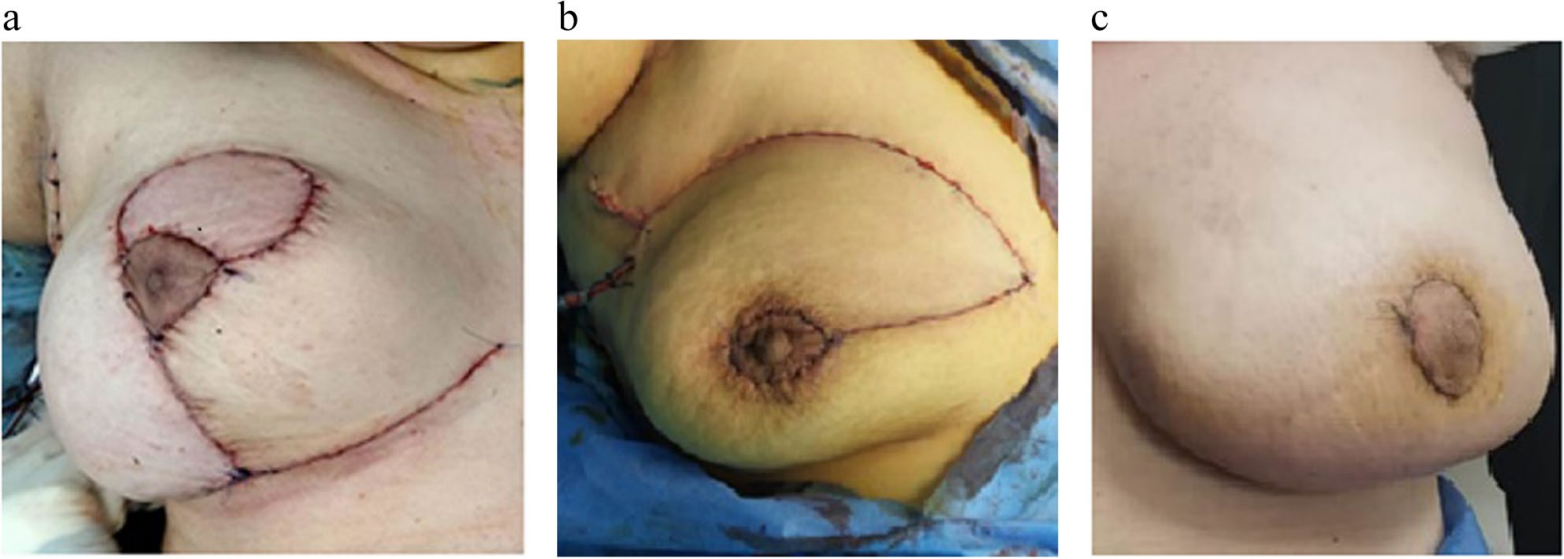
Showing the adopted three oncoplastic surgical techniques (**a**) inferior pedicle reduction mammoplasty, (**b**) upper matrix rotational flap and (**c**) Passot’s technique

Contralateral surgery was refused by most of our patients; we failed to get an informed consent for an immediate or delayed symmetrization Surgery (Fig. 1).

### Demographics and results

**Fig. 2:**
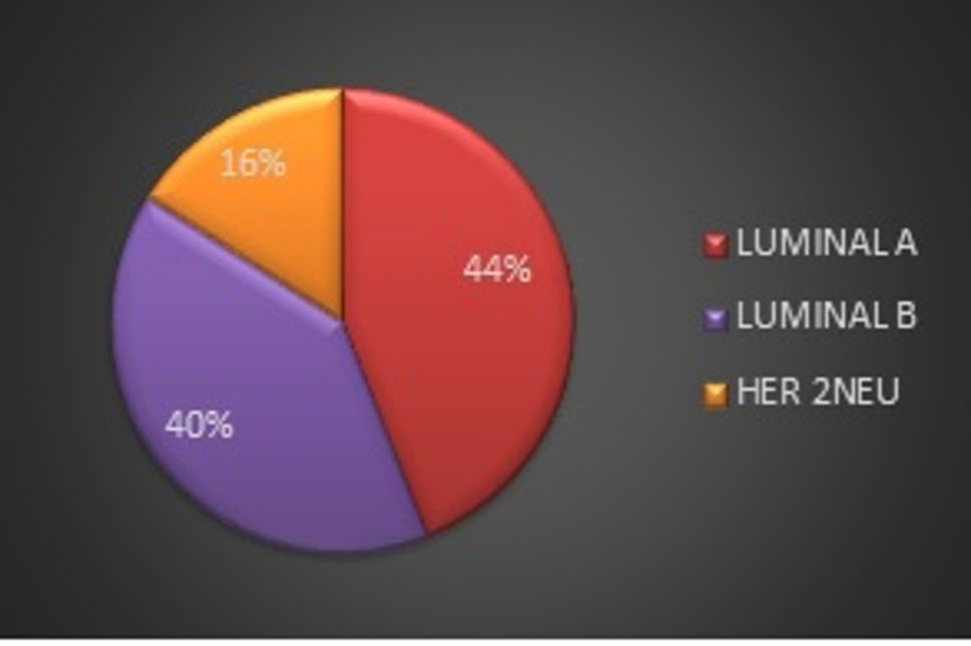
Showing the hormonal receptor status distribution among our patients

**Fig. 3:**
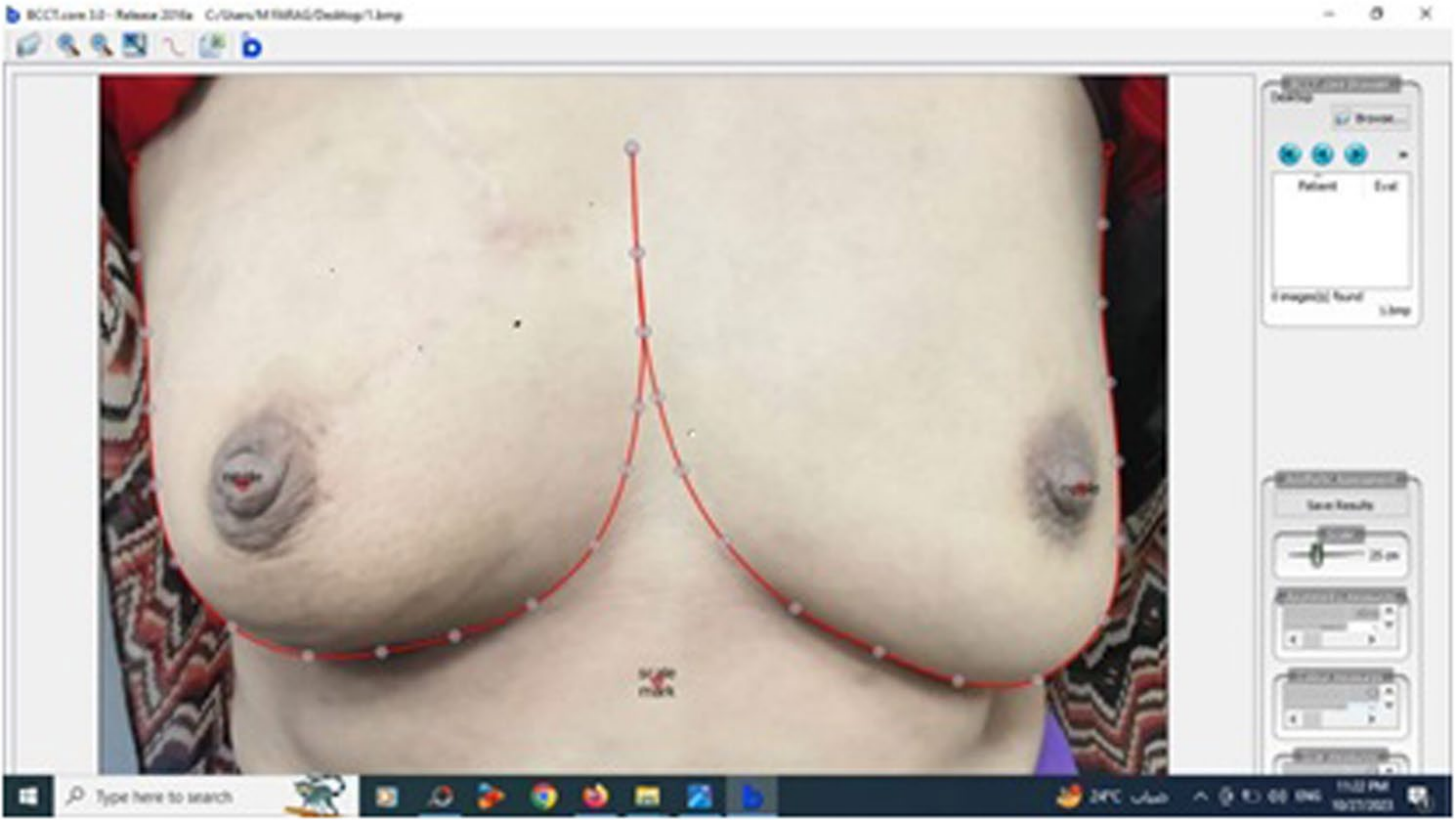
Extreme oncoplasty patient showing good BCCT core outcome

Fifty breast cancer patients fulfilling our eligibility criteria were included in the current study presented with invasive duct carcinoma (35 patients presented with tumour size more than 5 cm and 15 patients presented with multifocal disease). Our patients were 52.28 ± 6.3 years old on average, with a mean tumor size of 5.2 ± 0.38 cm and a mean resected volume of 353 ± 4 gm. Our aesthetic outcome was good in 52% (26 cases), fair in 34% (17 cases) and poor in 14% (7 cases). All of the excised specimens showed adequate safety margins with two reported cases of local recurrence after one year (4%) which were managed via mastectomy. We faced in the current study 20% complication rate among our patients (5 cases of skin bruises, 3 cases of wound dehiscence and 2 cases of wound infection). While skin bruises and wound infection were managed conservatively, wound dehiscence cases were managed via re-suturing (Fig. 2 and 3).

## Discussion

Breast cancer surgery has passed from an aggressive, disfiguring and bloody radical procedure with a high mortality rate to safe and aesthetically acceptable conservative counterparts with the same oncological outcomes and high survival rates by the work of some competent and ambitious surgeons who have created this amazing long path. Extreme oncoplasty was the harvest of this path which has provided the hope for the breast cancer patients who are in a need for mastectomy to preserve their breast with acceptable aesthetic outcome and oncological safety. Based upon the indications of the extreme oncoplasty, two important considerations should be taken into account. First, it is usually associated with higher tumour size and specimen volume which usually reflected on the aesthetic outcomes. The second issue is the narrower margins which usually reflected on the conversion rate to mastectomy.

The current study has showed an accepted aesthetic outcome in 86% (good and fair in BCCT core) which come compatible with the work of the other authors (aesthetic satisfaction rate from 78% to 83.5%) [12–16]. There are many different methods mentioned in literature for postoperative aesthetic assessment; Some rely on the subjective methods, which are the patient’s assessment or the observer’s assessment, while others rely on the objective methods, which are the physical and photographic measurements [17–21]. We have planned to rely on the objective approach (BCCT core) to review our aesthetic results, eliminating the possibility of subjective bias, in order to contribute to the ongoing effort to improve the cosmetic results of the extreme oncoplasty for our breast cancer patients and to obtain similar results.We reported a local recurrence rate of 4% which does not violate the mentioned recurrence rate in literature (up to 3.5%) and a mastectomy rate of 4% which come in correspondence to the mentioned rate for this from the previous works (up to 13.5%) [12, 22–24] The short duration of recurrence may be regarded to the high grade nature of most of our patients tumours. Silverstein et al. [25] working on 66 patients has declared in his work clear margins in 83.3% with (6.9%) as a re-excision rate and mastectomy was done for 6%. Authors demonstrated a varied complication rate for the extreme oncoplasty ranging from 7.7% to 28% [12, 13, 21, 23, 26]; we have faced in the same line a complication rate of 20% among our patients.

Since introduction of the extreme oncoplasty in literature, many questions has been raised around its results compared with the other counterparts techniques namely, breast implants and lipofilled flaps.

Breast implants has from 10 to 30% overall complication rate including Loss of natural sensation, skin necrosis, Rippling, Rupture, Capsular Contracture and Malposition. Those complications can be doubled with the detrimental effect of the adjuvant radiotherapy. Implant exclusion, cutaneous necrosis, aging of reconstruction.

Risk of those complications increases over time which stand against it as a lifetime method of reconstruction, this is another considerable point.

Also, due to higher local recurrence rate of extreme oncoplasty, postoperative strict follow up is an important issue. Breast implants mandate more demanding methods of investigations for follow-up like MRI and sometimes [27, 28].

Fat augmented latissimus dorsi flap or lipofilled mini dorsi flap can be an alternative to the extreme oncoplasty with added complications of donor site, fat necrosis, partial or total flap loss, volume loss and rare functional issues regarding range of shoulder joint motion with an overall complication rate of 5% to 20% [29].

## Conclusion

Over the last two decades, oncoplastic surgical techniques has allowed breast surgeons to perform more extensive excisions, preserve breast aesthetics and limit the indications of mastectomy. Extreme oncoplasty has broaden the indications of breast conservation for breast cancer patients with high oncological success rate, high rate of aesthetic satisfaction and lower rate of complications. Current study has revealed similar results for our patients using three of the advanced oncoplastic techniques. Those promising results mentioned in literature try to represent the extreme oncoplasty as a way out of the difficult situations in breast surgery.

## Data Availability

All data supporting the findings of this study are available within the paper the corresponding author has an excel sheet with the data upon request.
